# Meta‐analyzing the likely cross‐species responses to climate change

**DOI:** 10.1002/ece3.5617

**Published:** 2019-08-29

**Authors:** Jean C. G. Ortega, Nathália Machado, José Alexandre Felizola Diniz‐Filho, Thiago F. Rangel, Miguel B. Araújo, Rafael Loyola, Luis Mauricio Bini

**Affiliations:** ^1^ Programa de Pós‐Graduação em Ecologia e Evolução Universidade Federal de Goiás Goiânia Brazil; ^2^ Departamento de Ecologia Universidade Federal de Goiás Goiânia Brazil; ^3^ Brazilian Research Network on Climate Change – Rede Clima Instituto Nacional de Pesquisas Espaciais São José dos Campos Brazil; ^4^ Departamento de Biodiversidad y Biología Evolutiva Museo Nacional de Ciencias Naturales CSIC Madrid Spain; ^5^ Cátedra de Biodiversidade Rui Nabeiro Universidade de Évora Évora Portugal; ^6^ Center for Macroecology, Evolution and Climate University of Copenhagen Copenhagen Denmark; ^7^ Fundação Brasileira para o Desenvolvimento Sustentável Rio de Janeiro Brazil

**Keywords:** ecological niche modeling, global warming, meta‐analysis, range size, species distribution, uncertainty

## Abstract

Ecological Niche Models (ENMs) have different performances in predicting potential geographic distributions. Here we meta‐analyzed the likely effects of climate change on the potential geographic distribution of 1,205 bird species from the Neotropical region, modeled using eight ENMs and three Atmosphere‐Ocean General Circulation Models (AOGCM). We considered the variability in ENMs performance to estimate a weighted mean difference between potential geographic distributions for baseline and future climates. On average, potential future ranges were projected to be from 25.7% to 44.5% smaller than current potential ranges across species. However, we found that 0.2% to 18.3% of the total variance in range shifts occurred “within species” (i.e., owing to the use of different modeling techniques and climate models) and 81.7% to 99.8% remained between species (i.e., it could be explained by ecological correlates). Using meta‐analytical techniques akin to regression, we also showed that potential range shifts are barely predicted by bird biological traits. We demonstrated that one can combine and reduce species‐specific effects with high uncertainty in ENMs and also explore potential causes of climate change effect on species using meta‐analytical tools. We also highlight that the search for powerful correlates of climate change‐induced range shifts can be a promising line of investigation.

## Graphical abstract

Different ecological niche models can lead to divergent expectations of climate change impacts in species range distribution. We show that weighting by ecological niche model precision can help to improve cross‐species range changes predictions. The variability in cross‐species expected range changes is more due to differences among species than among ecological niche models, which highlights that searching for species trait that may predict range changes can help to target conservation.

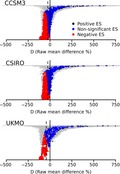

## 1 INTRODUCTION

Evidence supporting human‐induced global warming and its effects on biological processes and biodiversity patterns are accumulating conspicuously (Parmesan, 2006; Pecl et al., 2017). This evidence comes from multiple sources, including studies on phenology (e.g., time of leaf unfolding events), shifts in the border of species' geographic ranges (Parmesan & Yohe, 2003) and those based on controlled experiments (Walker et al., 2006). Empirical evidence of the effects of global warming on biodiversity is so overwhelming that the number of synthesis of these impacts is increasing steadily (e.g., Ainsworth, Rosenberg, & Wang, 2007 and references therein; Halupka & Halupka, 2017).

Following the broad‐scale analyses supporting human‐induced climate change, many recent studies evaluated the likely effects of climate change on the geographic range of several species belonging to large taxonomic groups in different biogeographic regions at variable spatial extents (Diniz‐Filho et al., 2009; Garcia, Burgess, Cabeza, Rahbek, & Araújo, 2012; Jetz, Wilcove, & Dobson, 2007; Lawler, Shafer, Bancroft, & Blaustein, 2010; Lawler et al., 2009; Sales, Neves, Marco, & Loyola, 2017; Terribile et al., 2012; Thuiller et al., 2011). These studies estimated potential range shifts by using one or several methods for niche modeling associated with different Atmosphere‐Ocean General Circulation Models (AOGCM) and Greenhouse Gas Emission Scenarios (GES). If one is interested in summarizing these results, in principle, the projected range shift of each species could be used as an unit of information in a meta‐analysis.

Different issues arise when applying formal meta‐analytic techniques to summarize niche modeling results, but critical is the uncertainty associated with range shift estimates. For example, Diniz‐Filho et al. (2009) showed that the estimated shifts in species' geographic ranges varied broadly, mainly due to the use of different Ecological Niche Models (ENMs hereafter). Other sources of uncertainty include the choices of AOGCM, GES, parameterization methods and rules to transform continuous outputs of models into presence and absence estimates (Nenzén & Araújo, 2011). In other words, different model projections are obtained when different combinations of ENMs, GES, and AOGCMs are used. Furthermore, the characteristics of the modeled organisms (e.g., life history and dispersal ability) can also influence model outcomes (Buisson, Thuiller, Casajus, Lek, & Grenouillet, 2010; Lawler et al., 2010; Thuiller, Guéguen, Renaud, Karger, & Zimmermann, 2019).

Here, we propose that the uncertainty of species' range shifts can be used as weights in a meta‐analysis. We exemplify our new approach using the estimated biogeographical range shifts of Neotropical birds to future climate change. Further, in a cross‐species analysis, we asked whether the weighted mean projected shift has a negative (loss) or a positive (gain of geographic range) signal. Finally, we tested the predictive ability of species‐specific biological traits as correlates of range shifts.

## 2 MATERIAL AND METHODS

### 2.1 Data

We exemplify our analytical framework with the dataset originally used by Diniz‐Filho et al. (2009). This dataset includes the extents of occurrence (range filling) for 1,205 Neotropical bird species. The data were downloaded from the BirdLife (former Nature Serve; http://www.datazone.birdlife.org) and resampled to a grid of 1° latitude × 1° longitude.

We used four bioclimatic variables in our ENMs (mean annual rainfall and variability, average temperature of the warmest and coldest months) because they represent the major drivers of species diversity in large spatial scales (see Hawkings, Porter, & Diniz‐Filho, 2003). We obtained data on bioclimatic variables from the World Climate Research Program's (WCRP) Coupled Model Intercomparison Project phase 3 (CMIP3) multimodel dataset (Meehl et al., 2007; https://www.esgf-node.llnl.gov).

We used a subset of the species (*n* = 1,205) modeled by Diniz‐Filho et al. (2009) because we kept only those for which the following variables were available (see Appendix S1): altitude midpoint, body size, IUCN categories of extinction risk, clutch size, and migratory behavior (migrants or non‐migrants). A previous study indicated that these traits are important in predicting bird extinction risk (Machado & Loyola, 2013). Altitude midpoint was defined as the mean between maximum and minimum altitude within ranges. Data on body size, clutch size, and migratory behavior came from different sources (see Machado & Loyola, 2013). IUCN categories of extinction risk were transformed to a discrete scale attributing zero, 1, 2, 3, and 4 to least concern (LC), near‐threatened species (NT), vulnerable species (VU), endangered species (EN), and critically endangered species (CR), respectively (Cardillo et al., 2004; Purvis, Gittleman, Cowlishaw, & Mace, 2000).

We built a majority rule consensus phylogeny (Bryant, 2003) among the species with 10,000 random phylogenetic trees with “Hackett constraint” for the backbone topology from Jetz, Thomas, Joy, Hartmann, and Mooers (2012). For matching, we used the function match.phylo.comm of “picante” (Kembel et al., 2010) and the function “consensus.edges” of “phytools” R package (Revell, 2012) to build the consensus phylogeny.

### 2.2 Statistical analyses

Our analyses were divided in three steps. First, for each species, we fitted eight ENMs (assuming unlimited dispersal): BIOCLIM, Euclidean distance, Random Forest, Generalized Additive Model, Generalized Linear Model, Gower distance, Multivariate Adaptive Regression Splines and Maxent (see Rangel & Loyola, 2012). Using these models, we estimated the potential geographic distributions for baseline and future climates, considering three AOGCM (CCSM3, CSIRO‐Mk3.0 (CSIRO), and UKMO‐HadCM3 (UKMO); see Diniz‐Filho et al., 2009 for details), for the scenario A1 (IPCC, 2000). In sum, for each species, we generated 24 values of potential geographic range size (eight ENMs projected into three AOGCMs), both for baseline and future climates (generating a total of 48 projections). The eight ENMs listed above were originally selected by Diniz‐Filho et al. (2009) to study the variation in the results derived from the use of different modeling strategies, from simple (e.g., BLIOCLIM) to more computer‐intensive methods (Random Forest). Because a key aspect of our approach also consists in measuring the variation in range shift and, for comparative purposes, we use the same ENMs.

Second, for each species *i*, within a given AOGCM and for each ENM *j*, we estimated the proportional difference between potential geographic distributions for future and baseline climates (*D_ij_*):

$$D_{\mathrm{ij}}=\frac{y_{\mathrm{ij}}-x_{\mathrm{ij}}}{x_{\mathrm{ij}}}\times 100$$

where *y_ij_* is the future potential geographic distribution (number of 1° × 1° grid cells) for species *i* according to the ENM *j*; *x_ij_* is the baseline potential geographic distribution (number of 1° × 1° grid cells) for the same species *i* and ENM *j*. A negative *D_ij_* value suggests that species *i* will lose a proportion in its projected range of occurrence according to ENM *j*. These proportional differences were then averaged across the eight ENMs, generating *D_i_*. The *D_i_* was taken as a measure of effect size in our study (difference in geographic distribution; henceforth “effect size” for simplicity and to adhere to the standard nomenclature for meta‐analysis). The uncertainty in the estimates was evaluated by the variance (*V_i_*) over the eight values of *D_ij_* for each species within a given AOGCM.

Third, the weighted mean range shift (*M*), summarizing the results across the 1,205 bird species, was estimated assuming a random‐effects model, where the weight (*W_i_*) assigned to each species *i* was given by the reciprocal of the *V_i_* plus the between‐species variance (*T* ^2^). In a typical meta‐analysis, *T* ^2^ is an estimate of the between‐studies variance in effect sizes (see Borenstein, Hedges, Higgins, & Rothstein, 2009; Borenstein, Higgins, Hedges, & Rothstein, 2017). In our study, this statistic reflects the variability among the species in terms of their likely responses to climate change (difference between current and future potential geographic distribution). Thus, the larger the variance (*V_i_*) associated to a *D_i_*, which, in our study, is due to the use of different ENMs, the smaller the weight of a species in estimating *M*. The larger the between‐species variance (*T* ^2^), the larger our uncertainty over *M*.

Our random‐effects model consisted of a multilevel model with bird phylogeny as a random effect (Lajeunesse, 2009; Nakagawa & Santos, 2012) and was estimated using the “rma.mv” function in the “metafor” R package (Viechtbauer, 2010). We estimated a phylogenetic variance‐covariance matrix describing the expected variance (in the diagonal) and the covariances (in the off‐diagonals) of a given trait following a Brownian‐motion process (Lajeunesse, 2009), using the “vcv” function of “ape” package (Paradis, Claude, & Strimmer, 2004). We used this phylogenetic variance‐covariance matrix to describe the correlation among species' responses in the random‐effects multilevel model.

We estimated a *T* ^2^ statistic of variability between species ($T_{s}^{2}$) and due to the phylogenetic random effects ($T_{p}^{2}$) with the multi‐level model (Nakagawa & Santos, 2012). We also estimated a statistic called *I* ^2^, which indicates the proportion of variability in the effect sizes (i.e., range shifts) that comes from true differences between species and, therefore, can likely be explained by species‐level variables. We further decomposed *I* ^2^ into two components: between‐species heterogeneity ($I_{s}^{2}$) and phylogenetic‐level heterogeneity ($I_{p}^{2}$; Nakagawa & Santos, 2012).

To illustrate the possibility of using meta‐analytic tools to explore the reasons for the variability in effect sizes (i.e., the among‐species variation in range shifts), we evaluated whether the magnitude of the effects sizes (*D_i_*) was related to the following set of variables: altitude midpoint, body size, species' IUCN categories of extinction risk, clutch size, and migratory behavior. For this, we applied random‐effects multilevel meta‐regression models, while controlling for phylogenetic effects (Lajeunesse, 2009; Nakagawa & Santos, 2012). For each meta‐regression model, we estimated a quantity analogous to the coefficient of determination (pseudo‐*R* ^2^) following Borenstein et al. (2009). All analyses were performed in the R environment (R Core Team, 2018) using the packages cited above (Appendix S2).

## 3 RESULTS

Projected mean range shifts varied conspicuously among species but were consistent across different AOGCMs. Nearly half (or more) of the 1,205 species had a projected decrease in their geographic range (CCSM3 = 509 species; CSIRO = 509 species; UKMO = 628 species). For species projected to suffer a reduction in range size, unweighted mean losses were 49.6% (*SD* = 16.2%), 49.7% (*SD* = 16.1%), and 72.4% (*SD* = 19.0%) of the current area under CCSM3, CSIRO, and UKMO AOGCMs, respectively.

**Figure 1:**
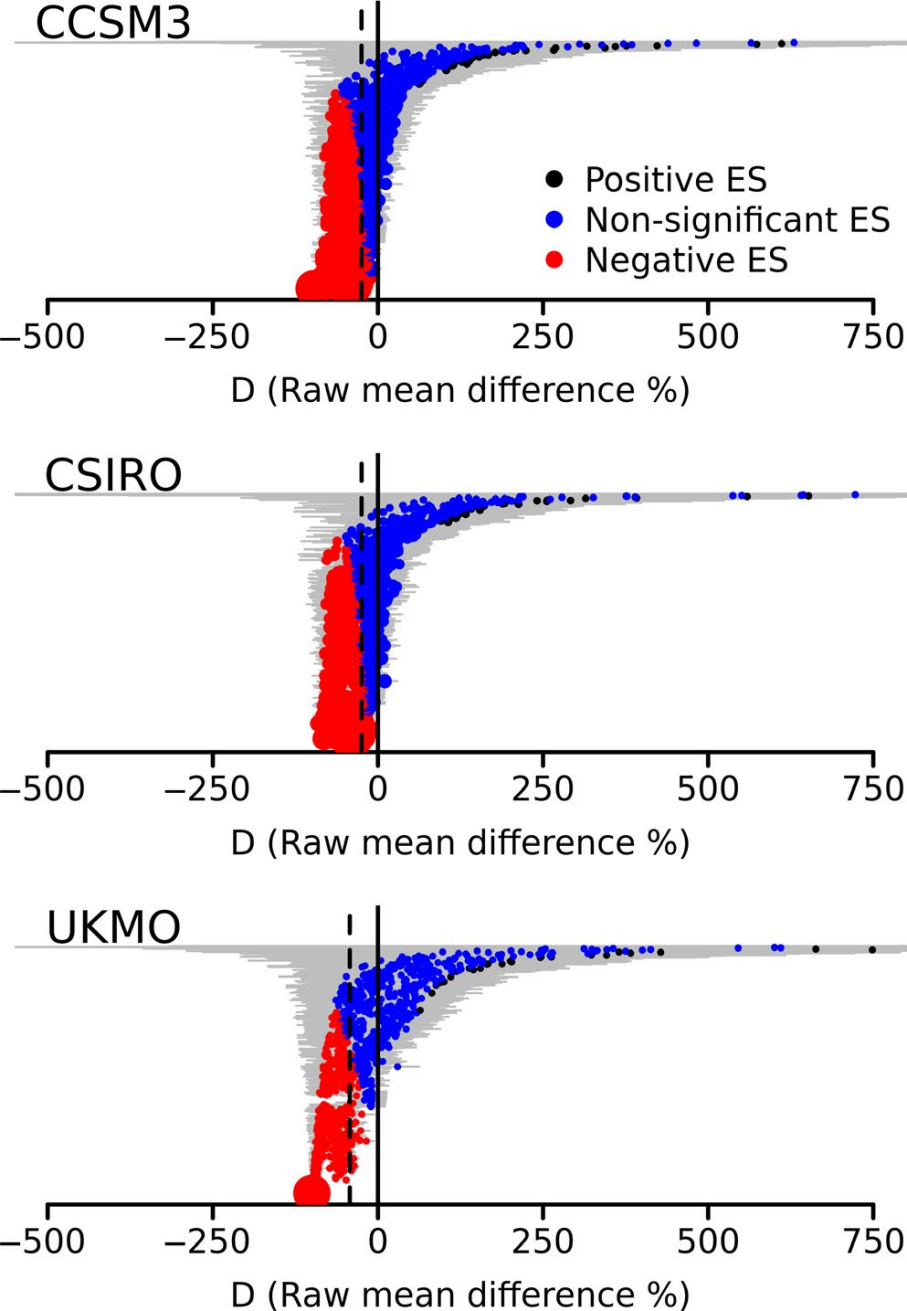
Variation of effects sizes among different atmospheric‐ocean global circulation models projections. The vertical solid line indicates effect size equal to zero, and the dashed line indicates the weighted effect size for each projection. Horizontal lines indicate 95% confidence intervals (95% CI) of each effect size. The size of each circle indicates the weight of each effect size for the weighted effect size calculation. Negative ES: effect sizes which the upper limit of 95% CI does not include zero; Positive ES: effect sizes which the lower limit of 95% CI does not include zero; Non‐significant ES: effect sizes which 95% CI includes zero. For simplicity, we omit six, five, and eight very imprecise effects sizes in “a”, “b,” and “c,” respectively

Another ca. 50% of species neither decreased nor increased their projected geographic range size (CCSM3 = 663 species; CSIRO = 668 species; UKMO = 547 species). Only a small fraction of species was projected to increase its geographic range size (CCSM3 = 33 species; CSIRO = 28 species; UKMO = 30 species), with mean increase of 344.8% (*SD* = 551.6%), 359.8% (*SD* = 587.4%), and 494.1% (*SD* = 788.1%) of the current area under CCSM3, CSIRO, and UKMO, respectively. Across all AOGCM, large range shifts tended to be less precise, showing wider confidence intervals (Figure 1).

The weighted mean effect size *M* (i.e., the difference between species potential distributions for baseline and future climates) was estimated to be −24.9% (95% CI = ±6.8%), −24.7% (±6.3%) and −42.6% (±17.2%) under CCSM3, CSIRO, and UKMO, respectively (Figure 1). There was significant heterogeneity in all AOGCM, and the highest values of the heterogeneity (*T* ^2^) was due to between‐species differences (Table 1). According to the coefficient $I_{t}^{2}$, from 81.3% to 99.8% of the observed variance can be attributable to real differences among species in terms of geographic range shift (*D_i_*). From 65.9% to 74.3% of the observed variance in *D_i_* can be due to real differences between species, and from 7.0% to 34.0% can be due to phylogenetic effects (Table 1). The high variability in the differences between potential distributions for baseline and future climates suggests that there is much scope for the study of correlates of range loss/gain due to climate change.

**Table 1:** Heterogeneity measures of effect size variation among atmospheric‐ocean global circulation models (AOGCM)

AOGCM	*Q*	*df*	$T_{s}^{2}$	$T_{p}^{2}$	$I_{t}^{2}$	$I_{s}^{2}$	$I_{p}^{2}$
CCSM3	6,561.6	1,204	514.9 (±22.7)	57.7 (±7.6)	82.2	73.9	8.3
CSIRO	5,794.4	1,204	500.6 (±22.4)	47.1 (±6.9)	81.3	74.3	7.0
UKMO	23,850.6	1,204	891.9 (±29.9)	459.6 (±21.4)	99.8	65.9	34.0

Note

All *Q* statistic were significant with *p* < .01. $T_{s}^{2}$: *T* ^2^ statistic for species random effects; $T_{p}^{2}$: *T* ^2^ statistic for phylogenetic random effects; $I_{t}^{2}$: total *I* ^2^ statistic; $I_{s}^{2}$: between species *I* ^2^ statistic; $I_{p}^{2}$: phylogenetic effects *I* ^2^ statistic. Standard errors of *T* ^2^ estimate are in parentheses.

**Figure 2:**
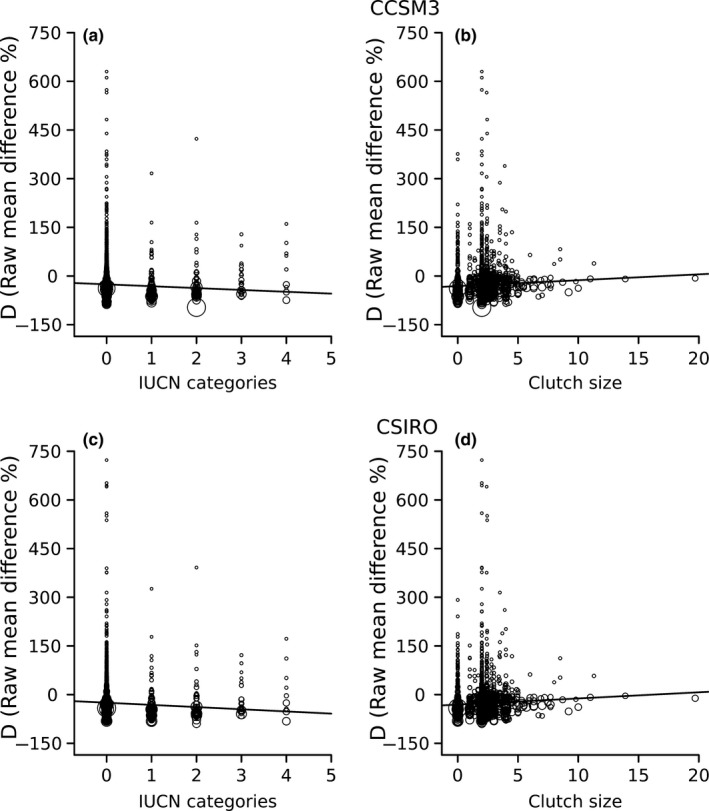
Relationship between raw mean difference (*D*, %) and IUCN categories of extinction risk (a, c) and clutch size (b, d). Top row indicates atmospheric‐ocean global circulation models projections of CCSM3, and bottom row CSIRO. Circle size indicates the weight of each effect size to meta‐regression parameter estimates. Fitted lines represent partial effects of each moderator variable on *D*

**Figure 3:**
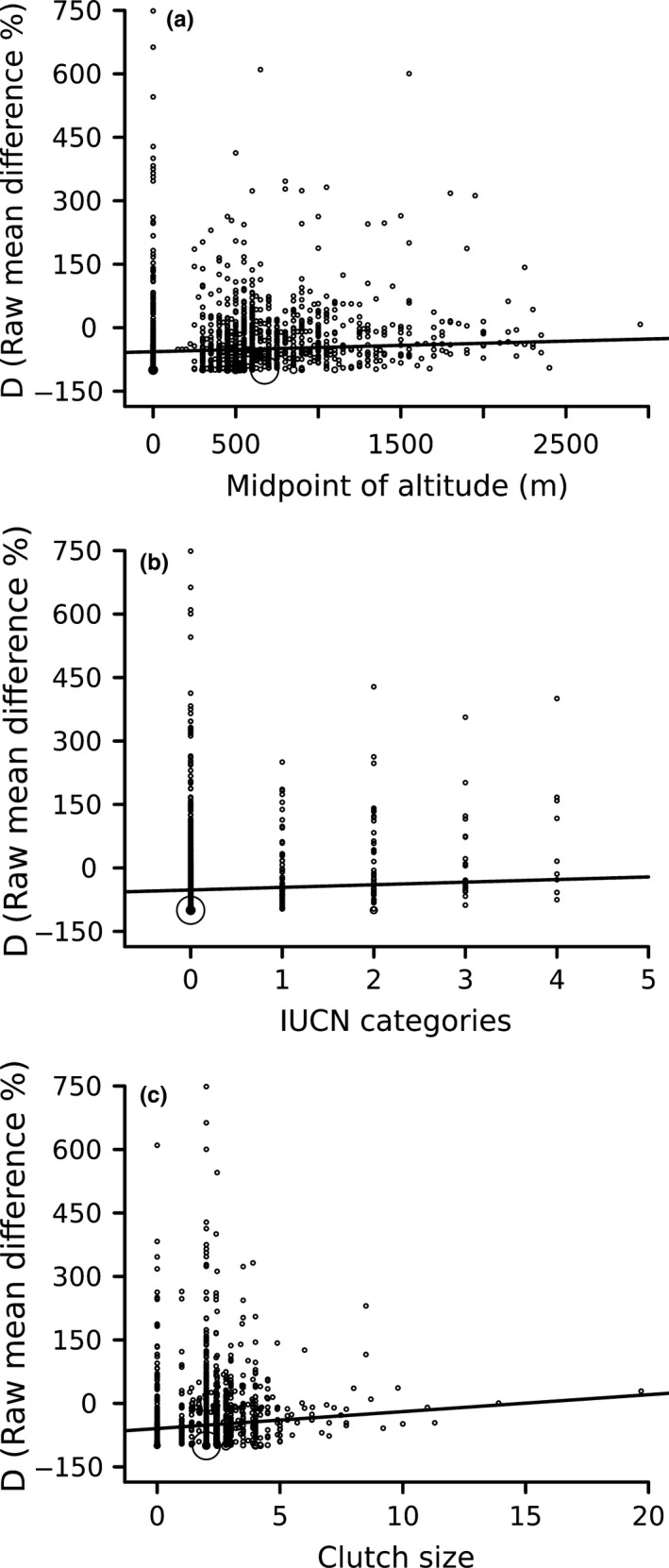
Relationship between raw mean difference (*D*, %) and species midpoint of altitude (a), IUCN categories of extinction risk (b) and clutch size (c) for atmospheric‐ocean global circulation models projections of UKMO. Circle size indicates the weight of each effect size to meta‐regression parameter estimates. Fitted lines represent partial effects of each moderator variable on *D*

Meta‐regression models had low predictive power (CCSM3: pseudo‐*R* ^2^ = 0.07; CSIRO: pseudo‐*R* ^2^ = 0.09; UKMO: pseudo‐*R* ^2^ = 0.12). IUCN categories of extinction risk, clutch size, and altitude midpoint (only for UKMO) were the main predictors of differences in species' range shifts (Table 2). The higher the risk of extinction the greater the reduction in range size under both CCSM3 and CSIRO climate models (Figure 2a,c). Further, species with larger clutch size tended to have lower reduction in range size under both CCSM3 and CSIRO (Figure 2b,d). Using UKMO, species occurring at higher altitudinal midpoint, extinction risk, and with larger clutch size had a lower reduction in range size (Figure 3).

**Table 2:** Meta‐regression parameter estimates among atmospheric‐ocean global circulation models (AOGCM)

AOGCM	Variable	Estimate	*SE*	95% CI_low_	95% CI_up_	*t*	*p*
CCSM3	Intercept	−34.66	7.10	−48.58	−20.73	−4.88	<.01
	Body size	0.0002	0.001	−0.001	0.002	0.23	.82
	Midpoint of altitude	0.0003	0.002	−0.003	0.004	0.15	.88
	IUCN categories	−5.71	1.32	−8.30	−3.13	−4.33	<.01
	Clutch size	1.81	0.64	0.55	3.06	2.82	<.01
	Migration_(Absence)_	4.65	6.06	−7.23	16.54	0.77	.44
CSIRO	Intercept	−39.36	6.71	−52.52	−26.19	−5.87	<.01
	Body size	0.000	0.001	−0.002	0.001	−0.04	.97
	Midpoint of altitude	−0.001	0.002	−0.005	0.002	−0.69	.49
	IUCN categories	−6.71	1.26	−9.18	−4.24	−5.33	<.01
	Clutch size	1.89	0.62	0.67	3.12	3.04	<.01
	Migration_(Absence)_	10.91	5.74	−0.35	22.18	1.90	.06
UKMO	Intercept	−80.94	12.55	−105.56	−56.32	−6.45	<.01
	Body size	0.001	0.001	−0.002	0.003	0.46	.65
	Midpoint of altitude	0.01	0.002	0.01	0.02	4.74	<.01
	IUCN categories	6.16	1.84	2.56	9.76	3.36	<.01
	Clutch size	4.00	0.90	2.23	5.78	4.43	<.01
	Migration_(Absence)_	13.87	9.19	−4.17	31.90	1.51	.13

Note

Comparisons to “migration” level were conducted with deviation from the reference level (presence).

Abbreviations: 95% CI low and up: lower and upper bound of 95% confidence interval; *SE*: standard error.

## 4 DISCUSSION

We examined the potential effects of projected climate change on the geographic range sizes of Neotropical birds. Matching results obtained by many studies (Parmesan & Yohe, 2003; Walther et al., 2002; see Parmesan, 2006 for a comprehensive review), which measured phenological (e.g., advancement of spring events) and distributional changes (e.g., poleward shifts in geographical ranges, range contractions or expansions), we projected that effects of climate change on the range sizes of Neotropical birds can be negative. The negative sign of this forecasted pattern seems to be coherent (sensu Parmesan, 2006; Pecl et al., 2017) for the scale of the Neotropics. Further, we showed that more than 80% of the observed variance in species‐range size change can be attributable to real differences between species and, therefore, can potentially be explained by species‐levels (explanatory) variables. However, finding correlates of range shift proved to be difficult.

Even under the unrealistic assumption of unlimited dispersal (which favors the hypothesis of no effect of climate change on the biota), we found that 509 (for CCSM3 and CSIRO) to 628 species (for UKMO) from all 1,205 bird species assessed are projected to experience range contraction. Further, species that were projected to lose less or even to increase their range of occurrence tended to present the most imprecise estimates. Our estimates of the relative number of bird species suffering more than 50% range contractions (4.4%, 3.7% and 27.8% of all bird species considered, respectively for CCSM3, CSIRO, and UKMO) are also comparable to those made by Jetz et al. (2007), who found that from 4.5% to 20.6% of the species analyzed are likely to lose more than 50% of their ranges.

However, an important message of our study is that we should not attribute the same weight for different species when assessing cross‐species differences in potential distributions. This is so because, in the context of ensemble forecasting (Araújo & New, 2007), different ENMs provide different forecasts of potential distribution for the same species (e.g., Buisson et al., 2010; Diniz‐Filho et al., 2009; Garcia et al., 2012; Lawler et al., 2009; Rangel & Loyola, 2012; Sales et al., 2017; Thuiller, 2004). Our results considered these differences because we down‐weighted species with variable range shifts estimates due to the use of different ENMs. The reason behind weighting is that species with low uncertainties in terms of range shifts will give estimates of the effects of climate change on geographic ranges that are more robust to the choices of ENMs. Also, weighting the estimates of range shift accordingly will likely increase the likelihood of correctly estimating the potential effects of climate changes. If we did not weight the *D_i_* values, we would observe a mean difference in potential distribution of 6.38% (95% CI = ±12.44%), 6.28% (±12.49%) and −4.63% (±16.88%) under CCSM3, CSIRO, and UKMO projections, respectively. Thus, without taking uncertainty into account, our estimates of the effect of climate change on geographic range size would be much smaller and, probably, downward biased. In general, we believe that taking uncertainty into account, while estimating the effects of climate change on species distributions, will be an important step in rebutting climate change denialism (e.g., Boussalis & Coan, 2016).

Our meta‐analytic approach provides an alternative method to quantify the relative variation in estimates of range shifts within (i.e., due to the use of different ENMs and AOGCMs) and among species. Our results are in line with those reported previously by showing that a substantial part of the variation in range shifts (from 25.7% up to 34.1%) can be attributed to the use of different ENMs (for a recent study, see Thuiller et al., 2019). Further, the geographic range losses, as given by the weighted mean effect sizes (*M*), were estimated to be much higher (ca. 57%) under UKMO than under CSIRO. However, our findings also highlight that most of the variation in range shifts, for a given AOGCM, can be attributable to real differences between species and that a small part of this variation is phylogenetically structured (except under UKMO). Taken together, our results suggest that the main question (or source of uncertainty) is not whether many Neotropical bird species will lose a large proportion of their climatic space (as it has also been projected for northern‐boreal land bird; see Virkkala, Heikkinen, Leikola, & Luoto, 2008), but “which species will be under most threat” (Sinclair, White, & Newell, 2010).

The search for correlates of range shifts under contemporary climate change has been an active research line in ecology (e.g., MacLean & Beissinger, 2017; Williams & Blois, 2018). The method we used to decompose the variation in range shifts among species also allowed us to infer that phylogenetic relatedness is a poor predictor of range shifts (but see Comte, Murienne, & Grenouillet, 2014 for a contrasting result with stream fishes). We found that bird species with larger clutch size are expected to lose a smaller range of occurrence than species with smaller clutch size. This pattern was consistent across all AOGCM and with studies showing that species with higher fecundity may be buffered against a decrease in their overall range of occurrence (e.g., Amano & Yamamura, 2007). Bird species with higher threat of extinction (higher IUCN categories of extinction risk values) may lose a larger range of occurrence projected for CGCM and CSIRO. For UKMO, these species were projected to lose a smaller range of occurrence than species classified with a smaller extinction risk. Altitudinal midpoint correlated positively with differences in range of occurrence. However, studies have shown that mountain bird species will lose a greater range of occurrence than other species (e.g., La Sorte & Jetz, 2010; Freeman, Scholer, Ruiz‐Gutierez, & Fitzpatrick, 2018; see also a meta‐analysis by Scridel et al., 2018). A possible reason for the difference between our results and those from other studies is that our dataset included only 30 species (2.5% from all bird species we considered) with distributions restricted to high altitudes (following the classification proposed by Stotz, Fitzpatrick, Parker, & Moskovits, 1996; e.g., *Accipter striatus* Vieillot, 1808, *Microcerculus ustulatus* Salvin & Godman, 1883, *Roraimia adusta* (Salvin & Godman, 1884), and *Trogon personatus* Gould, 1842). This unbalance toward species with larger range size and not restricted to high altitudes may be the reason for the positive relationship between midpoint of altitude and difference in potential geographic distribution. Therefore, this result should be interpreted with caution, also considering that it was detected under UKMO only. In general, our results suggest that the choice of AOGCMs is also an important aspect to be considered in studies searching for correlates of range shift.

Finally, had we assumed dispersal limitation in our ENMs, our estimates of range shift would be even greater (depicting a more pessimistic scenario) because dispersal limitation would make it difficult for species to track their optimal conditions in space (Hof & Allen, 2019; Wang, He, Thompson, Spetich, & Fraser, 2018). One could also argue that our estimates of potential geographic range loss would be lower after allowing for adaptation. However, recent studies indicate that the effect of adaptation (evolutionary rescue) may be limited (Cotto et al., 2017; Diniz‐Filho et al., 2019). Valladares et al. (2014) showed that the forecasted effects of climate change on species range shifts are expected to even increase when phenotypic plasticity is incorporated into ENMs. Our estimates of variation in range shifts are likely to be conservative because a recent study by Thuiller et al. (2019) showed that ENMs differ greatly in predicting future distributions even when only ENMs with high predictive accuracies are considered in the analyses.

Despite the uncertainties associated with ENMs, which were considered in the meta‐analytic approach implemented here, our results suggest that the effects of human‐induced global warming on the geographic distributions of Neotropical birds are concerning. We believe that a promising line of investigation would consist in finding other species traits with high capability to predict likely ranges shifts (as forecasted by ENMs). For instance, an additional bird trait not considered here is the extent of breeding seasons. In a recent meta‐analysis, Halupka and Halupka (2017), for example, found that climate change may extend breeding seasons for multi‐brooded species and reduce it for single‐breeding species from the northern hemisphere. Species reproducing for a longer period may increase their population size and consequently the range that this species occurs. Finding strong correlates of range shift would help policymakers to focus not only where the effects of climate changes are likely to be stronger (e.g., Jetz et al., 2007; Lawler et al., 2010; Lawler et al., 2009), but also to list the species (as predicted by their traits) that are at higher risk.

## CONFLICT OF INTEREST

None declared.

## AUTHOR CONTRIBUTIONS

LMB, RL, TFR, JAFDF, and MBA designed the idea. NM, TFR, JCGO collected data and performed analyses. JCGO and LMB wrote the first draft. All authors contributed to the elaboration of the final version.

## Data Availability

All the data and script used are available at the Dryad Digital Repository (https://doi.org/10.5061/dryad.qv5p3r8).
